# Transient Focal Cerebral Ischemia Leads to miRNA Alterations in Different Brain Regions, Blood Serum, Liver, and Spleen

**DOI:** 10.3390/ijms23010161

**Published:** 2021-12-23

**Authors:** Clara Voelz, Nahal Ebrahimy, Weiyi Zhao, Pardes Habib, Adib Zendedel, Thomas Pufe, Cordian Beyer, Alexander Slowik

**Affiliations:** 1 Institute of Neuroanatomy, Medical Faculty, RWTH Aachen University, 52074 Aachen, Germany; cvoelz@ukaachen.de (C.V.); nahal.ebrahimy@ukbonn.de (N.E.); 15663661681@163.com (W.Z.); azendedel@ukaachen.de (A.Z.); cbeyer@ukaachen.de (C.B.); 2 Department of Neurology, Medical Faculty, RWTH Aachen University, 52074 Aachen, Germany; phabib@ukaachen.de; 3 JARA-BRAIN Institute of Molecular Neuroscience and Neuroimaging, Forschungszentrum Jülich GmbH, RWTH Aachen University, 52074 Aachen, Germany; 4 Department of Anatomy and Cell Biology, Medical Faculty, RWTH Aachen University, 52074 Aachen, Germany; tpufe@ukaachen.de

**Keywords:** tMCAo, miRNA regulation, CNS, organ crosstalk, intercellular transfer

## Abstract

Ischemic stroke is characterized by an occlusion of a cerebral blood vessel resulting in neuronal cell death due to nutritional and oxygen deficiency. Additionally, post-ischemic cell death is augmented after reperfusion. These events are paralleled by dysregulated miRNA expression profiles in the peri-infarct area. Understanding the underlying molecular mechanism in the peri-infarct region is crucial for developing promising therapeutics. Utilizing a tMCAo (transient Middle Cerebral Artery occlusion) model in rats, we studied the expression levels of the miRNAs (miR) 223-3p, 155-5p, 3473, and 448-5p in the cortex, amygdala, thalamus, and hippocampus of both the ipsi- and contralateral hemispheres. Additionally, the levels in the blood serum, spleen, and liver and the expression of their target genes, namely, *Nlrp3*, *Socs1*, *Socs3*, and *Vegfa*, were assessed. We observed an increase in all miRNAs on the ipsilateral side of the cerebral cortex in a time-dependent manner and increased miRNAs levels (miR-223-3p, miR-3473, and miR-448-5p) in the contralateral hemisphere after 72 h. Besides the cerebral cortex, the amygdala presented increased expression levels, whereas the thalamus and hippocampus showed no alterations. Different levels of the investigated miRNAs were detected in blood serum, liver, and spleen. The gene targets were altered not only in the peri-infarct area of the cortex but selectively increased in the investigated non-affected brain regions along with the spleen and liver during the reperfusion time up to 72 h. Our results suggest a supra-regional influence of miRNAs following ischemic stroke, which should be studied to further identify whether miRNAs are transported or locally upregulated.

## 1. Introduction

Acute ischemic stroke (AIS) is characterized by an occluded blood vessel that results in an infarct core—a brain region that is not sufficiently perfused with oxygen and nutrients, leading to irreversible cell death. The adjacent brain region, called penumbra (or peri-infarct area), is metastable and might undergo apoptotic cell death. The latter is potentially salvageable and is a therapeutical target for promising novel therapies. However, with time, secondary damage such as oxidative stress and inflammation leads to further cell death and expansion of the infarct core [1]. Additionally, reperfusion injuries might occur after restoring the blood flow that amplifies the damage [2]. One detail of these events is dysregulated microRNAs (miRNAs) with various functions in protein expression control [3]. For this reason, it is necessary to understand the processes following the acute event to prevent prolonged cell death.

A miRNA consists of about 22 nucleotides (nt) with a seed region of about 8 nt [4]. Their regulation is altered upon injuries and illnesses, and they are investigated firstly due to their potential as biomarkers and secondly because they can regulate the expression of their target proteins [5,6]. By complementary binding to mRNAs, miRNAs inhibit the translational process and regulate the cell’s proteome [7,8]. miRNAs are found within a specific cell and can be released and subsequently detected in body fluids such as the blood plasma [9] or cerebrospinal fluid [10,11]. Previous studies have reported that miRNAs can be absorbed and perform translational repression in other cells than their origin [12,13,14]. The transfer is not limited to the same cell type or tissue that the exosomes enter but can be paracrine. The exosomes enter the cells by different mechanisms, e.g., via endocytosis. However, whether an miRNA-specific signal is involved remains elusive to date [15,16]. Besides active release processes, passive release by cell leakage is also possible. If the miRNA is bound to AGO2 or specific lipids, it is protected from degradation when not packed into exosomes.

Since discovering miRNAs, their regulation has been studied intensively after various injuries and diseases, including ischemic stroke. Therefore, many miRNA arrays have been performed with very different results, showing the high variance of this method, which might be due to sample type and preparations and different ways of animal surgeries performed [17,18,19,20].

In several studies before, researchers tried to identify specific miRNAs as biomarkers in the bloodstream to monitor the stage of an ischemic infarct in patients. Based on that idea, we hypothesized that altered miRNAs after an ischemic insult would also be detectable in different non-affected brain regions showing different expression patterns. A step further, in a second hypothesis, we assumed that altered expression patterns of these miRNAs could also be found in peripheral organs, such as the liver and spleen. For this study, we focused on four miRNAs (223-3p, 155-5p, 3473, and 448-5p) that we found regulated following AIS and were also detectable in all tissues of interest. Furthermore, these four miRNAs are known to target proteins involved in the inflammatory reaction following AIS.

For miR-223-3p, a lot of research regarding the NLRP3 (NLR Family Pyrin Domain Containing 3) inflammasome has been conducted since NLRP3 seems to be negatively regulated by it [21,22,23]. Additionally, other pro-inflammatory cytokines and the pro-inflammatory phenotype of macrophages and neutrophils are attenuated by an increased expression of miR-223-3p [24]. In comparison, miR-155-5p seems to fulfill pro-inflammatory regulation by, e.g., increasing the expression of inflammatory cytokines, targeting suppressor of cytokine signaling 1 (SOCS1), dual-specificity phosphatase 14 (DUSP14), or antioxidant-related genes leading to reactive oxygen species production [25,26,27,28]. Both miRNAs were also described for many more target genes related to other cellular pathways besides inflammation. On the contrary, miR-3473 was barely studied until now. As well as connected to inflammation, miR-3473 seems to target TRAF3 (TNF receptor-associated factor 3), a negative regulator of the NF-κB pathway [29].

Additionally, SOCS3 seems to be a direct target of miR-3473b, as shown in a study of tMCAo [30]. Both studies on miR-3473 were conducted in mice and the miRNA-target interaction should be confirmed in rats to ensure the same regulation in a different organism. The last miRNA, miR-448-5p, was also scarcely studied until now. One study found VEGFA (vascular endothelial growth factor A) to be a target and a regulator of the FAS/FAS-L signaling pathway in a hypoxia model in myocardial rat cells [31]. VEGFA is known for angiogenesis and neuroprotection but seems to have a dual role since it alters vascular permeability and blood–brain barrier integrity in stroke [32,33].

In the end, we provide evidence that post-ischemic miRNA levels are altered in the stroke area and in regions that are not primarily affected, such as the contralateral brain, liver, and spleen. Likewise, target proteins show an alteration in different brain regions and distant tissues following AIS. These results lay the groundwork for future studies regarding organ-cross talk and miRNA signaling.

## 2. Results

**Figure A1:**
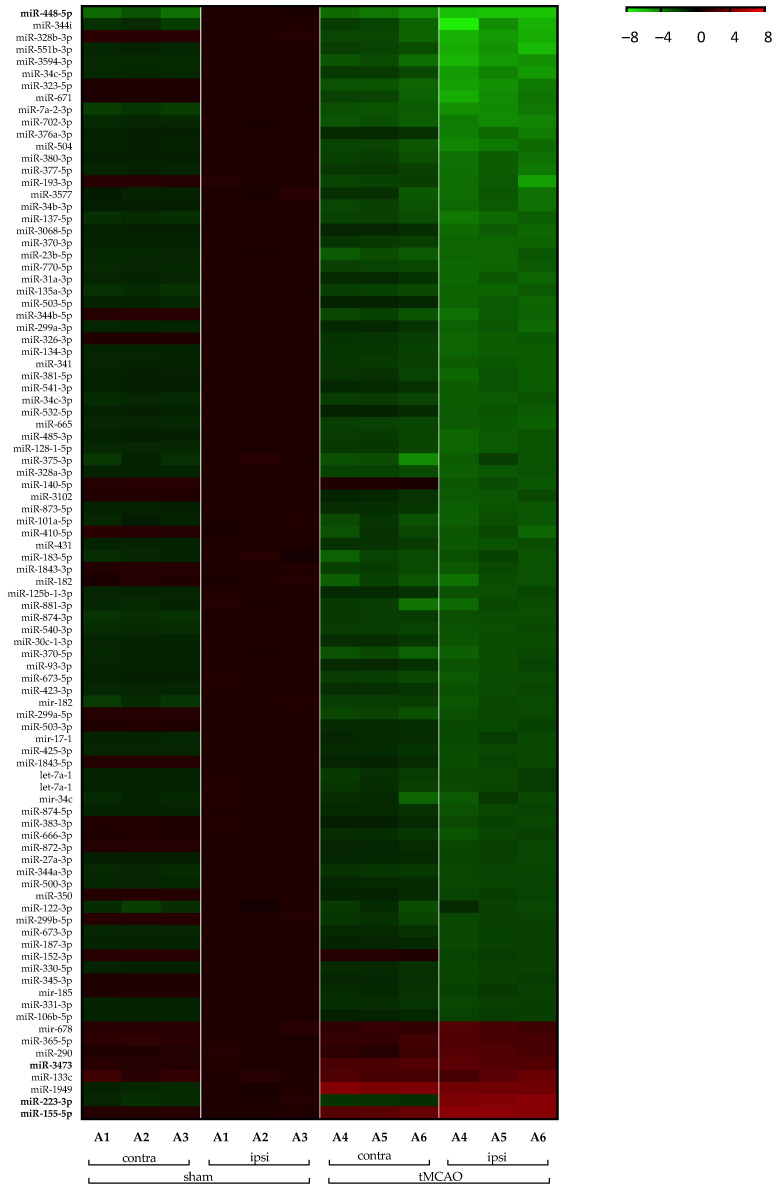
MicroRNA profiling using Affymetrix miRNA 4.0 Arrays. miRNAs with a fold-change of at least 1.5 compared to sham ipsi were considered differentially expressed. The heatmap was designed to show a 2-fold cut-off. Rows were normalized relative to the median. miRNA alteration is expressed from dark red (downregulated) to bright blue (upregulated). *n* = 3.

**Figure A2:**
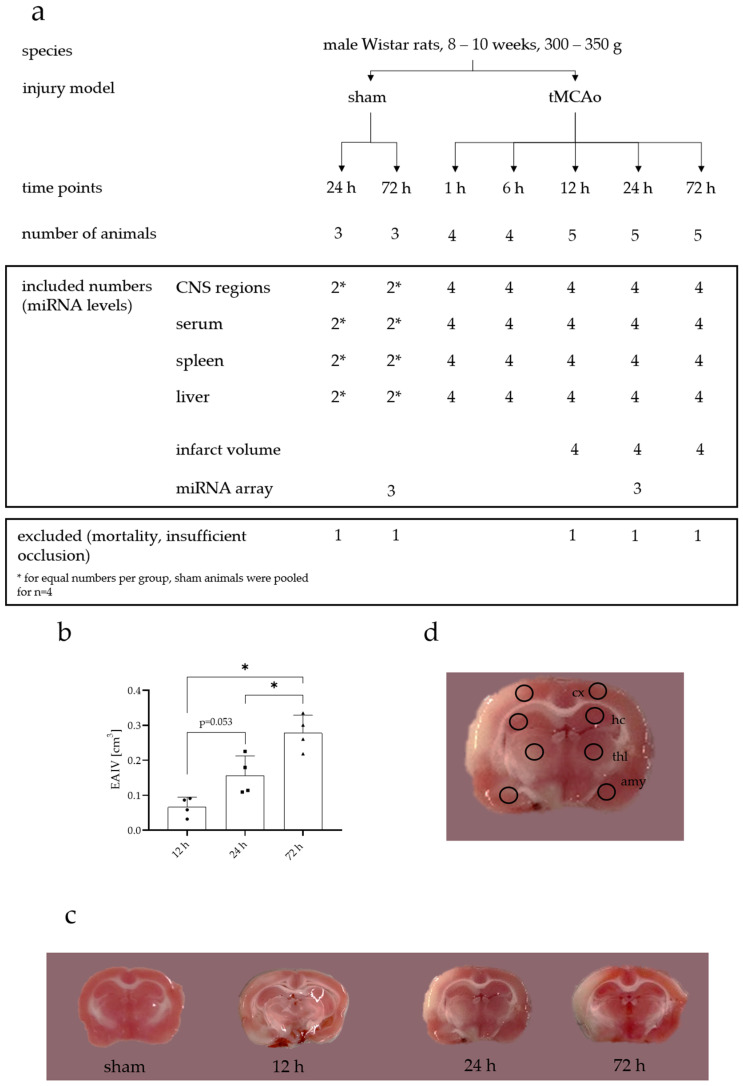
Animal numbers, infarct volume, and sampling location. To verify a sufficient stroke injury, we measured the infarct volume. (**a**) Animal numbers used in the study. (**b**) Edema-adjusted infarct volume (EAIV) in cm^3^ for all time points. For the 1 h and 6 h time points, a TTC staining is unreliable; therefore, this was not evaluated [95]. (**c**) Exemplarily shown brain slides after TTC staining. Necrotic regions are visible in white and show the infarct region, while healthy tissue is red. (**d**) Exemplarily shown brain slides with visible sites of punched sample collection. (cx) cortex, (hc) hippocampus, (thl) thalamus, (amy) amygdala. (*) shows a significant difference between the ipsilateral side and sham ipsi.

**Figure A3:**
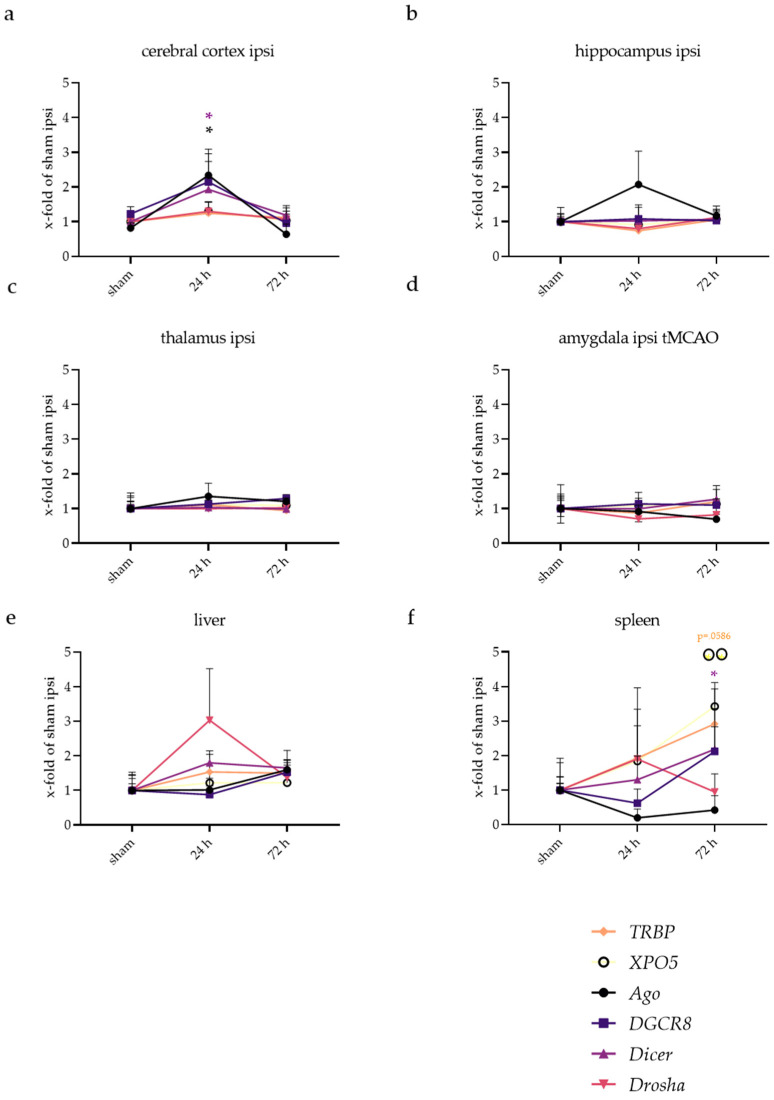
Expression of genes regarding miRNA maturation in different brain regions, liver, and spleen at 24 h and 72 h following tMCAo. (red) mRNA levels of *Drosha*, (dark purple) *DGCR8*, (yellow) *XPO5*, (light purple) *Dicer*, and (orange) *TRBP* in (**a**) the cerebral cortex, (**b**) amygdala, (**c**) thalamus, (**d**) hippocampus, (**e**) liver, and (**f**) spleen. (*) shows a significant difference from sham ipsi in the respective color of the gene. The statistics were performed in a group comparison with ipsi- and contralateral values. Here shown, there are just the alterations for the ipsilateral brain side.

In a first step, we evaluated the alteration in miRNAs after AIS. Therefore, miR-223-3p, miR-155-5p, miR-3473, and miR-448-5p were chosen, and their expression in different body tissues and fluids was determined. These miRNAs showed promising regulation results in a screening array, performed 24 h after tMCAo (Figure A1). Occlusion of the middle cerebral artery (MCA) for 60 min resulted in an increased infarct volume throughout the experiment with $\bar{x}$ = 0.27 cm^3^ after 72 h (Figure A2). We also studied genes involved in miRNA maturation and function (Figure A3) but found very little regulation, i.e., an upregulation of *Ago* and *Dicer* after 24 h in the cortex and an increase in *Dicer, XPO5,* and *TRBP* in the spleen after 72 h.

### 2.1. miRNA Expression Levels Were Mainly Increased on the Ipsilateral Side of the Cerebral Cortex

**Figure 1:**
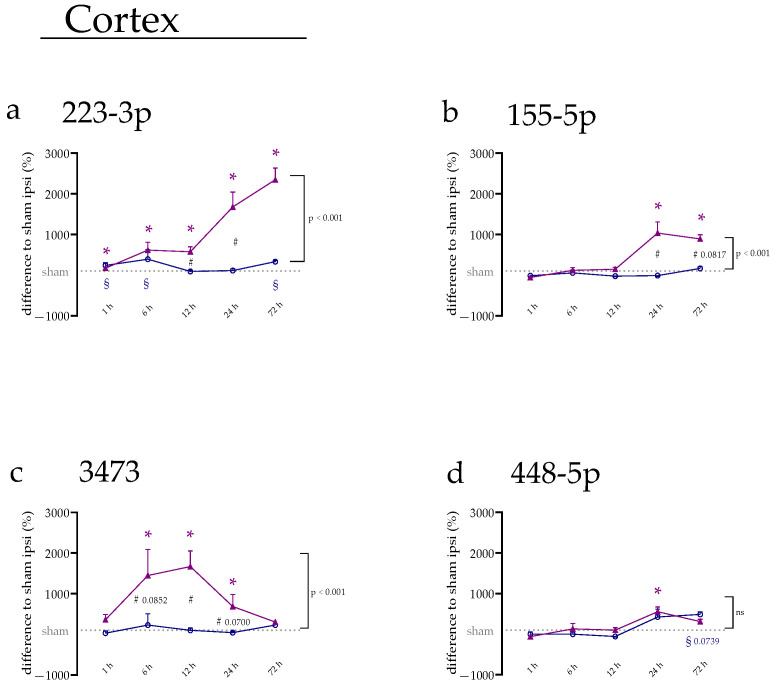
miRNA alteration in the cerebral cortex during the first three days after tMCAo. (**a**) miRNA levels of miR-223-3p, (**b**) miR-155-5p, (**c**) miR-3473, and (**d**) miR-448-5p of ipsi- (purple) and contralateral (blue) side of the cerebral cortex. Data are depicted as the difference in percentage (%) compared to the sham ipsi group ± SEM. (*) shows a significant difference between the ipsilateral side with sham ipsi; (#) shows a significant difference in the comparison of ipsi and contra at the specific time point; (§) shows a significant difference between the contralateral side with sham contra. Additionally, indicated on the side of the graph, there is the group difference between the ipsi- and contralateral hemispheres over the whole experimental set-up as calculated by two-way ANOVA.

To evaluate the miRNA expression after tMCAo, rat brains were cut into slices and stained with TTC to visualize the infarct regions. Thereby, we found that the infarct volume increased over time, as shown in Figure A2. Alterations in the expression were observed for the four selected miRNAs over time in the cerebral cortex for the ipsi- (brain hemisphere of the infarct side) and contralateral (control hemisphere without infarct) region. In Figure 1, the alteration in miRNA expression levels in the cerebral cortex is shown compared to sham. During the early reperfusion period, miR-223-3p increased on both sides (adjusted *p*-values at 1 h: ipsi, *p* < 0.001; contra: *p* = 0.0021; at 6 h: ipsi, *p* < 0.001; contra, *p* = 0.0018) when compared to the respective sham group (Figure 1a). After that, the expression on the ipsilateral side kept increasing throughout the experiment (highest expression about 24-fold at 72 h), while the contralateral side decreased again at 12 h (*p* = 0.5175) and 24 h (*p* = 0.3172). Here, the ipsi- and contralateral sides showed significant differences (ipsi vs. contra: 12 h, *p* = 0.0069; 24 h, *p* < 0.001) compared to each other. After 72 h, a significant increase in expression on the contralateral side was again observed (*p* < 0.001) and no statistical side differences could be found (ipsi vs. contra, *p* = 0.1523). Therefore, the expression of miR-223-3p depends on both factors, the reperfusion time and the brain allocation (ANOVA interaction, *p* < 0.001).

For miR-155-5p, no significant changes on the contralateral side were detected (Figure 1b). In contrast, an increase in miR-155-5p levels was observable after 24 h (*p* < 0.001) and after 72 h (*p* < 0.001) on the ipsilateral side in the cortical peri-infarct area. A significant difference between ipsi and contra was only visible at 24 h (*p* < 0.001), the time point with the highest miRNA expression measured (around 11-fold). The two-way ANOVA revealed a significant influence of reperfusion time and brain side on the expression of miR-155-5p (ANOVA interaction, *p* < 0.001).

Similar to miR-155-5p, no significant changes on the contralateral side were detected for miR-3473 (Figure 1c). On the ipsilateral side, the expression increased after 6 h (*p* = 0.0043) with the expression peak after 12 h (around 18-fold, *p* < 0.001), before it decreased again after 24 h (*p* = 0.0157) back to the baseline level at 72 h (*p* = 0.3289). At 12 h, the increase on the ipsilateral side led to a statistical difference between the ipsi- and contralateral brain hemispheres (*p* = 0.0122). The expression of miR-3473 was dependent on the reperfusion time and brain side (ANOVA interaction, *p* = 0.0259).

For miR-448-5p, the expression on the ipsi- and contralateral sides behave in a very similar way, though the expression on the contralateral side did not significantly increase during the whole experiment (Figure 1d). On the ipsilateral side, a 6.5-fold increase was observed of the miRNA expression at 24 h (*p* = 0.0406), which decreased again after 72 h (*p* = 0.5217). The two-way ANOVA revealed that the expression of miR-448-5p was only dependent on the different time points (*p* < 0.001), not the specific brain side.

### 2.2. miRNA Levels Were Increased after 24 and 72 h in the Cerebral Cortex and Amygdala

**Figure 2:**
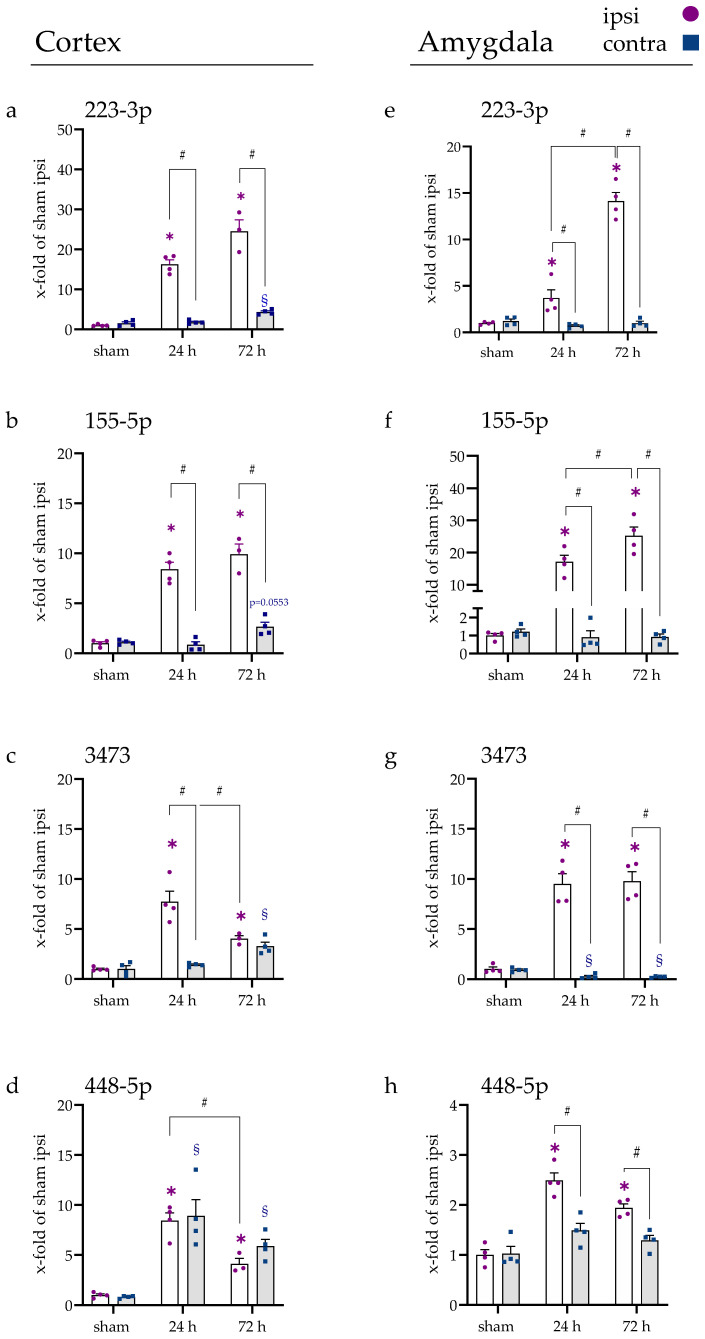
miRNA expression in cerebral cortex and amygdala after 24 h and 72 h following tMCAo. (**a**,**e**) miRNA levels of miR-223-3p in the cerebral cortex and amygdala, (**b**,**f**) miR-155-5p, (**c**,**g**) miR-3473, and (**d**,**h**) miR-448-5p. The graphs show the individual values for ipsi (purple) and contra (blue) and the mean in bars. Data are compared to the sham ipsi group and pictured with the x-fold expression of this group ± SEM. (*) shows a significant difference between the ipsilateral side and sham ipsi where (§) shows a significant difference between the contralateral side and sham contra. (#) comparison as indicated.

**Figure 3:**
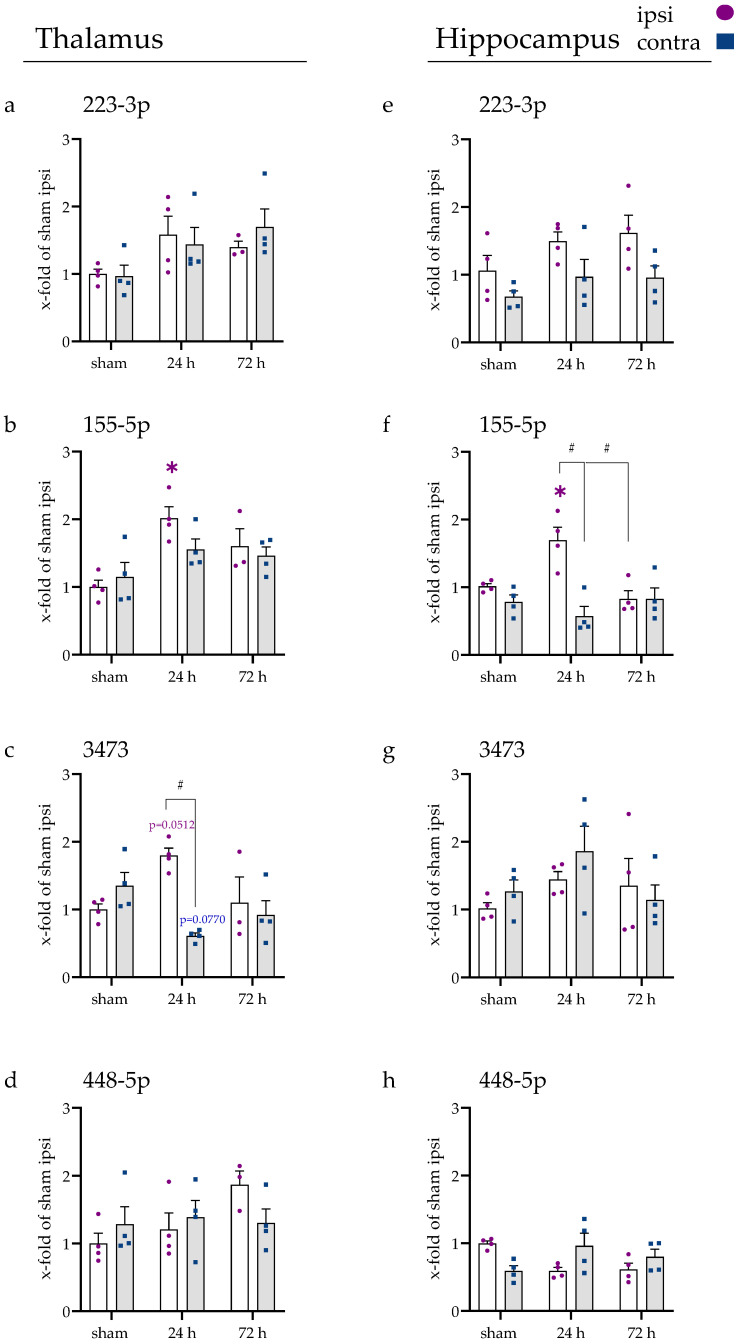
miRNA expression of thalamus and amygdala after 24 h and 72 h following tMCAo. (**a**,**e**) miRNA levels of miR-223-3p in thalamus and hippocampus, (**b**,**f**) miR-155-5p, (**c**,**g**) miR-3473, and (**d**,**h**) miR-448-5p. The graphs show the individual values for ipsi (purple) and contra (blue), and the mean in bars. Data were normalized to sham ipsi group and are pictured with the x-fold expression of this group ± SEM. (*) shows a significant difference between the ipsilateral side and sham ipsi. (#) comparison as indicated.

**Figure A4:**
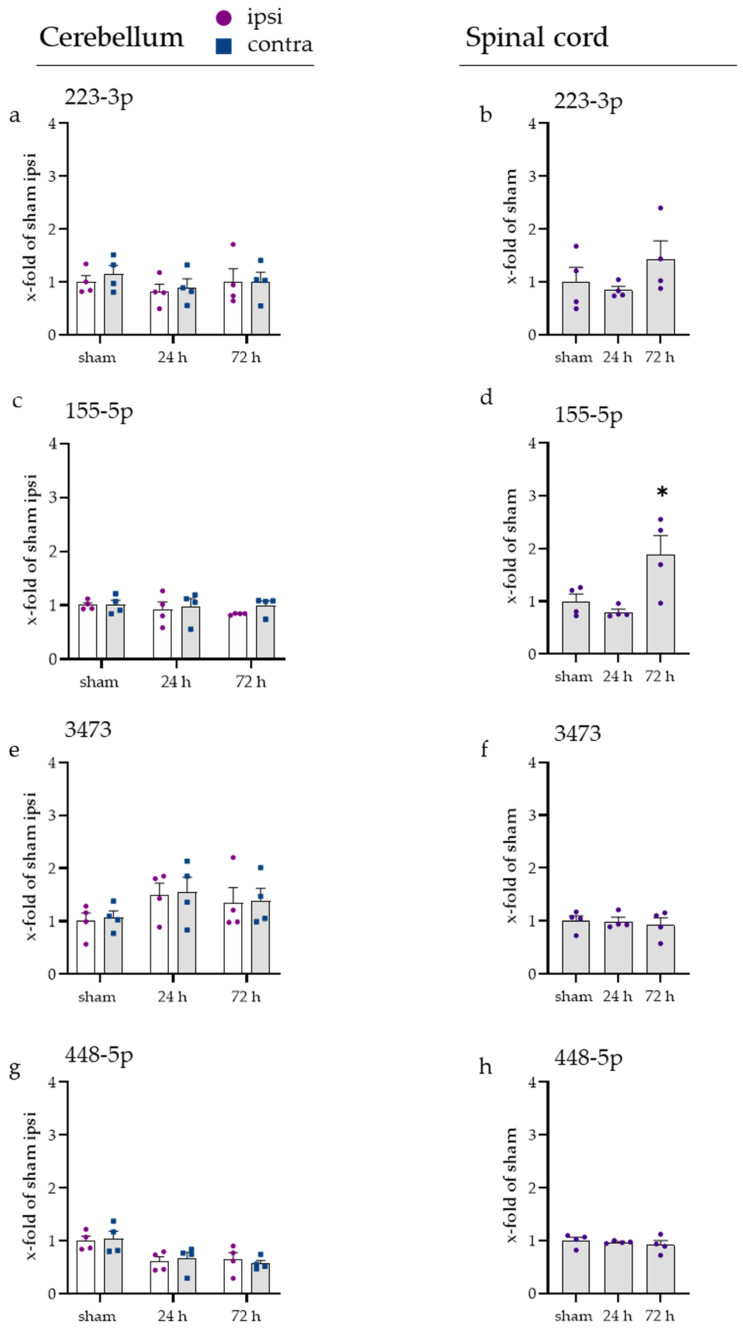
miRNA expression of the cerebellum and spinal cord after 24 h and 72 h following tMCAo. (**a**,**e**) miRNA levels of miR-223-3p, (**b**,**f**) miR-155-5p, (**c**,**g**) miR-3473, and (**d**,**h**) miR-448-5p. The graphs show the individual values for (purple) ipsi and (blue) contra and the mean in bars. Data were compared to the sham ipsi group and pictured with the x-fold expression of this group ± SEM. (*) shows a significant difference between the ipsilateral side and sham ipsi.

For the distribution of miRNAs during the later time points of 24 h and 72 h, the miRNA gene expression for cortex (Figure 2a–d), amygdala (Figure 2e–h), thalamus (Figure 3a–d), hippocampus (Figure 3e–h), cerebellum (Figure A4a–d), and spinal cord (Figure A4e–h) was considered more specifically. Cerebellum and spinal cord are not directly supplied by the MCA; therefore, they were not directly affected by the occlusion. No alterations of the miRNAs were observed in these two parts of the CNS, except for a 1.8-fold increase in the miR-155-5p gene expression after 72 h in the spinal cord (adjusted *p*-value, 0.0488).

In the cortex (Figure 2), miR-223-3p (a), miR-155-5p (b), miR-3473 (c), and miR-448-5p (d) were increased on the ipsilateral side at both time points (all *p* < 0.001) and on the contralateral side after 72 h (miR-3473 and miR-448-5p, *p* < 0.001; miR-223-3p, *p* = 0.0016; miR-155-5p, *p* = 0.0553). Only for miR-448-5p, the expression on the contralateral side was also significantly elevated after 24 h (*p* < 0.001). For miR-223-3p and miR-155-5p, the difference between ipsi and contra was significantly increased for both time points (*p* < 0.001). For miR-3473, the difference of ipsi and contra was measurable after 24 h (*p* < 0.001), but not after 72 h (*p* = 0.8780), due to an ipsilateral decrease in miR-3473 (24 h vs. 72 h, *p* = 0.0085) and a contralateral increase (24 h vs. 72 h, *p* = 0.0240). For miR-448-5p, no difference between ipsi and contra was observable at either time point (*p* > 0.45).

In the amygdala (Figure 2e–h), the expression of all four miRNAs was increased significantly at both time points on the ipsilateral side (all *p* < 0.001). Additionally, the expression increased from 24 h to 72 h for miR-223-3p (*p* < 0.001) and miR-155-5p (*p* = 0.0215).

The contralateral side showed a significant decrease in miR-3473 after 24 h and 72 h (both *p* < 0.001). Besides, no alteration in any other measured miRNA expression was detected. For all miRNAs and time points in the amygdala, the ipsi vs. contra expression revealed a significant difference (all *p* < 0.05). Note that the overall increase in miR-448-5p expression was lower in the amygdala compared to the other miRNAs.

In the thalamus (Figure 3a–d), the expression of miR-223-3p and miR-448-5p did not change in any of the studied occasions. After 24 h, a significant elevation was detected for miR-155-5p (*p* = 0.0056) and miR-3473 (*p* = 0.0512) on the ipsilateral side. On the contralateral side, only miR-3473 was slightly decreased (*p* = 0.0770) but only significantly different from its ipsilateral counterpart (*p* = 0.0021).

In the hippocampus (Figure 3e–h), no alteration in the expression of miR-223-3p, miR-448-5p, and miR-3473 was observed. For miR-155-5p, an elevation in expression was detected after 24 h compared to sham on the ipsilateral side (*p* = 0.0235), which lowered again after 72 h (24 h vs. 72 h, *p* = 0.0032). The comparison of ipsi and contra after 24 h tMCAo revealed a significant difference (*p* < 0.001).

### 2.3. miRNAs Fluctuated in Blood Serum over Time

**Figure 4:**
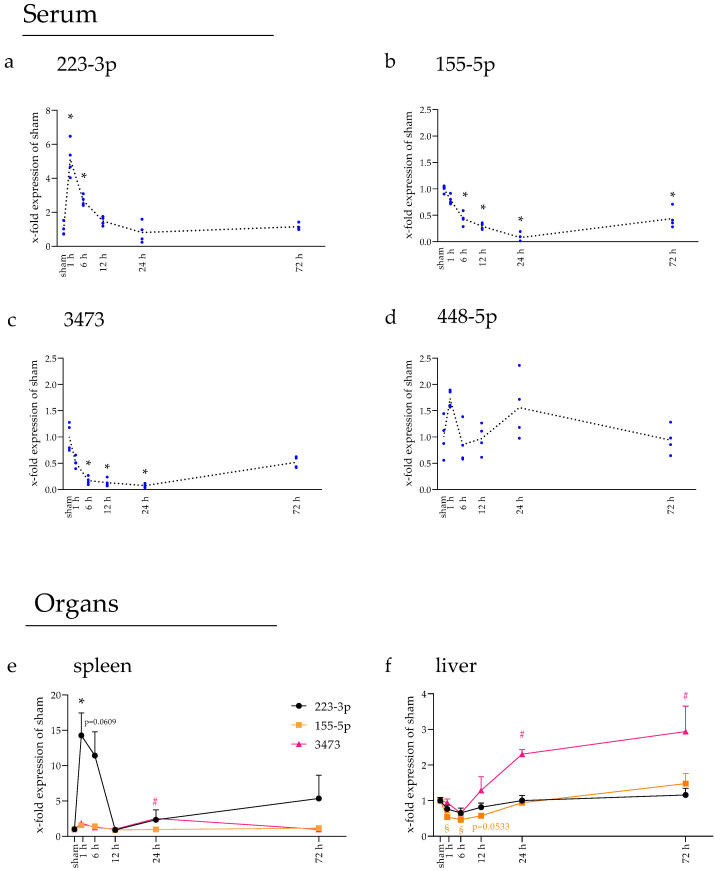
miRNA expression in blood serum and distant organs. (**a**) miRNA levels in serum for miR-223-3p, (**b**) miR-155-5p, (**c**) miR-3473, and (**d**) miR-448-5p. Individual values are pictured in the graphs. (*) shows a statistical significance between the expression at the specific time point and sham ipsi. (black) miRNA expression for miR-223-3p, (yellow) miR-155-5p, and (pink) miR-3473 in (**e**) the spleen and (**f**) liver was calculated separately but is shown in a combined graph. Note that miR-448-5p was not detectable in distant organs. In (**e**,**f**), (*) shows a significant difference for miR-223-3p, (§) for miR-155-5p, and (#) for miR-3473.

In the next step, we were interested in whether the investigated miRNA levels in the blood serum were also affected by the occlusion injury. Therefore, RNA was isolated and retrotranscribed and gene studies were performed using RT-qPCR. All four miRNAs were measurable in the blood serum and regulated, except for miR-448-5p (Figure 4a–d). The latter showed minor variances without reaching any statistical significance.

For miR-223-3p expression, a 5.1-fold increase was detected after 1 h compared to sham (adjusted *p*-value, <0.001), which decreased significantly at 6 h (1 h vs. 6 h, *p* < 0.0001) and returned to the baseline level at 12 h (*p* = 0.8085). miR-155-5p showed a steady decrease (all *p* < 0.001 past 6 h), with its lowest expression point at 24 h (around 7 % of sham expression). At 72 h, the expression increased again (24 h vs. 72 h, *p* = 0.0025). Similarly, miR-3473 expression decreased, with its lowest point at 24 h (around 7 % of sham expression), reaching a significant difference at 6 h after tMCAo (all indicated *p* < 0.001). After 72 h, the expression increased again significantly (24 h vs. 72 h, *p* < 0.0001), so no alteration was observable when compared to sham (*p* = 0.2358).

### 2.4. Spleen and Liver Responded to tMCAo with Increased miRNA Expression

To observe if the tMCAo injury could also lead to an alteration in the studied miRNAs in the spleen and liver, RNA was isolated and gene expression studies were conducted.

In the spleen, upregulation of miR-223-3p expression was observable after 1 h (adjusted *p*-value, 0.0107) and 6 h (*p* = 0.0609), which significantly decreased after 12 h (6 h vs. 12 h, *p* = 0.0482) and never reached a significant threshold again (all following time points > 0.9). The expression of miR-3473 increased after 24 h compared to sham (*p* = 0.0125) and decreased again after that (24 h vs. 72 h, *p* = 0.0108). No alteration in miR-155-5p expression could be observed in the spleen.

In the liver, the expression of miR-155-5p was found to be down-regulated after 1 h (*p* = 0.0264), 6 h (*p* = 0.0033), and 12 h (*p* = 0.0533) compared to sham. At 24 h and 72 h, the expression was found unaltered. For miR-3473, the gene expression increased significantly after 24 h (*p* = 0.0281) and 72 h (*p* = 0.0076) compared to sham (12 h vs. 24 h, *p* = 0.0512). No alteration in the gene expression of miR-223-3p was found at any time point in the liver. In both organs, miR-448-5p was not detectable at all.

### 2.5. Inflammatory Responses Also Visible in Injury-Remote Areas

**Figure 5:**
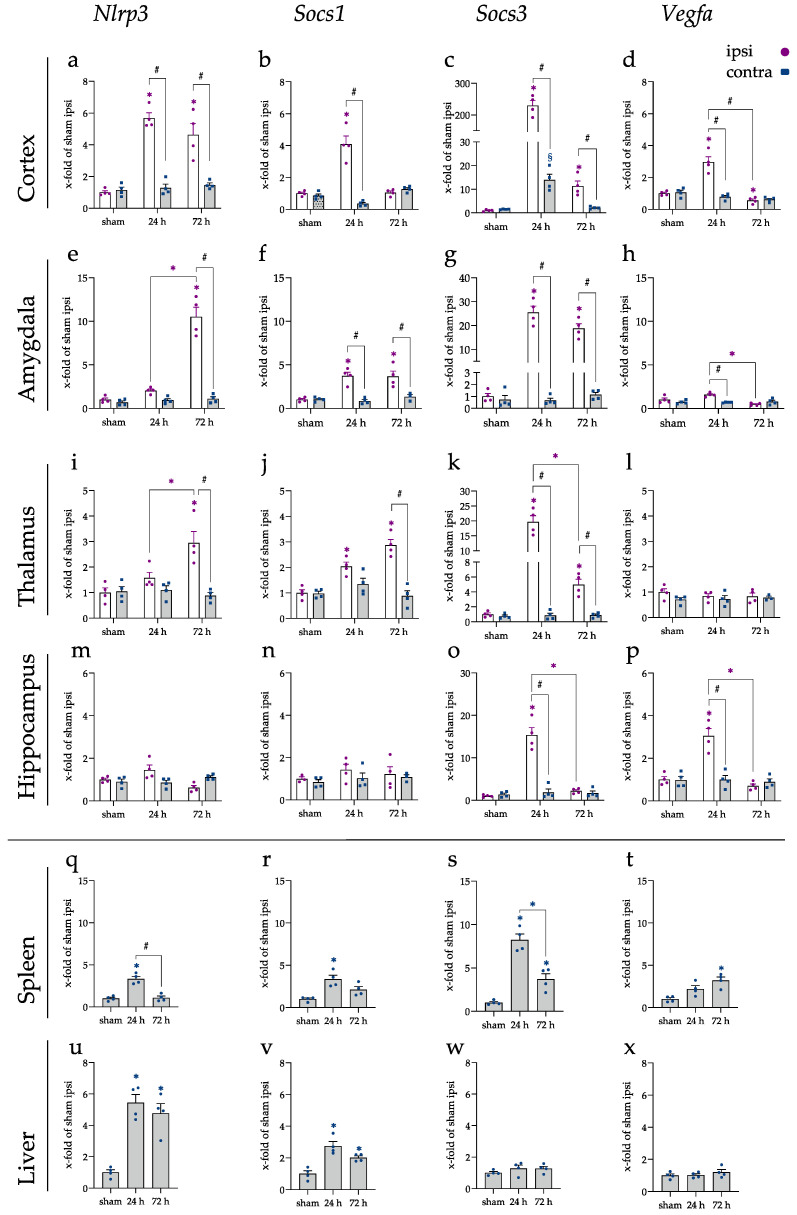
mRNA levels of *Nlrp3, Socs1, Socs3, and Vegfa.* The mRNA levels were measured in different brain parts and the peripheral organs, spleen, and liver. The results are sorted in rows for the various tissues and columns for the individual target. (**a**–**p**) results of different brain areas and (**q**–**x**) results of liver and spleen. The graphs show the individual values for ipsi (purple) and contra (blue) and the mean in bars. Data were normalized to sham ipsi group and are pictured with the x-fold expression of this group ± SEM. (*) shows a significant difference between the ipsilateral side and sham ipsi, where (§) shows a significant difference between the contralateral side and sham contra. (#) comparison as indicated.

Utilizing qRT-PCR, *Nlrp3, Socs1, Socs3,* and *Vegfa genes* were measured in the cortex (Figure 5a–d), amygdala (Figure 5e–h), thalamus (Figure 5i–l), and hippocampus (Figure 5m–p), but also in the spleen (Figure 5q–t) and liver (Figure 5u–x). For *Nlrp3,* the gene expression in the cerebral cortex was elevated after 24 and 72 h in the ipsilateral brain hemisphere (*p* < 0.001). In the amygdala and thalamus, gene expression rose ipsilateral after 72 h, leading to a significant difference compared to sham and the contralateral brain hemisphere (all *p* < 0.001). The hippocampus displayed no level changes for *Nlrp3*. In the peripheral organs, spleen, and liver, on the other hand, significantly increased levels of *Nlrp3* were found. In the spleen, an increase was visible after 24 h (*p* < 0.001), which was back at baseline after 72 h. *Nlrp3* levels remained elevated in the liver after 72 h (*p* < 0.001).

The mRNA levels of *Socs1* were found elevated in the cerebral cortex after 24 h on the ipsilateral side (*p* < 0.001). *Socs1* displayed significantly increased mRNA levels in the amygdala at both time points (*p* < 0.001). In addition, in the thalamus, at 24 h (*p* = 0.010) and 72 h (*p* < 0.001) after the injury, *Socs1* levels were significantly elevated with a significant difference between the brain hemispheres at 72 h (*p* < 0.001). In addition, for *Socs1,* no alteration could be found within the hippocampus.

On the other hand, the levels of *Socs3* were found elevated in all investigated brain areas. In the cortex, mRNA increased over 200-fold in the cerebral cortex ipsilateral and 14-fold in the cerebral cortex contralateral, resulting in a significant difference in respect to sham for both brain hemispheres (both *p* < 0.001). After 72 h, the increase in the contralateral hemispheres was reversed, while *Socs3* was still significantly increased ipsilateral (*p* < 0.001). At both time points, the alteration between the sides remained significantly different (both *p* < 0.001). *Socs3* mRNA levels were increased in the amygdala and thalamus at both time points in the ipsilateral brain hemisphere (all *p* < 0.001). In the hippocampus, a 15-fold increase on the ipsilateral side of *Socs3* led to a significant difference compared to sham and the contralateral brain hemisphere (both *p* < 0.001).

For *Vegfa*, the cortex and hippocampus displayed an increase after 24 h (both *p* < 0.001), while no alteration in the amygdala (*p* = 0.05) or thalamus were found in comparison to the sham group. Additionally, the mRNA levels decreased after 72 h in the cerebral cortex compared to sham (*p* = 0.04) and the amygdala compared to 24 h (*p* < 0.001; sham, *p* = 0.09).

In the spleen and liver, alterations in *Nlrp3, Socs1, Socs3*, and *Vegfa* were also observed. *Nlrp3* levels increased significantly in the spleen after 24 h (*p* < 0.001) but were back at baseline at 72 h. In the liver, the levels were increased at both experimental time points (both *p* < 0.001). A similar pattern was observed for *Socs1*, whereby an elevation in the spleen after 24 h (*p* = 0.0025) and at both time points in the liver was observed (24 h, *p* < 0.001; 72 h, *p* = 0.0168). For *Socs3,* no alteration was visible in the liver. In the spleen, an 8-fold increase in mRNA was observed after 24 h (*p* < 0.001), which was decreased after 72 h, still with a significant elevation when compared to sham (*p* = 0.0161). *Vegfa* displayed a significant 3-fold increase only after 72 h in the spleen (*p* = 0.0031).

### 2.6. Brain-Enriched miRNAs miR-328b-3p and miR-344i Were Not Found in the Spleen or Liver

**Figure A5:**
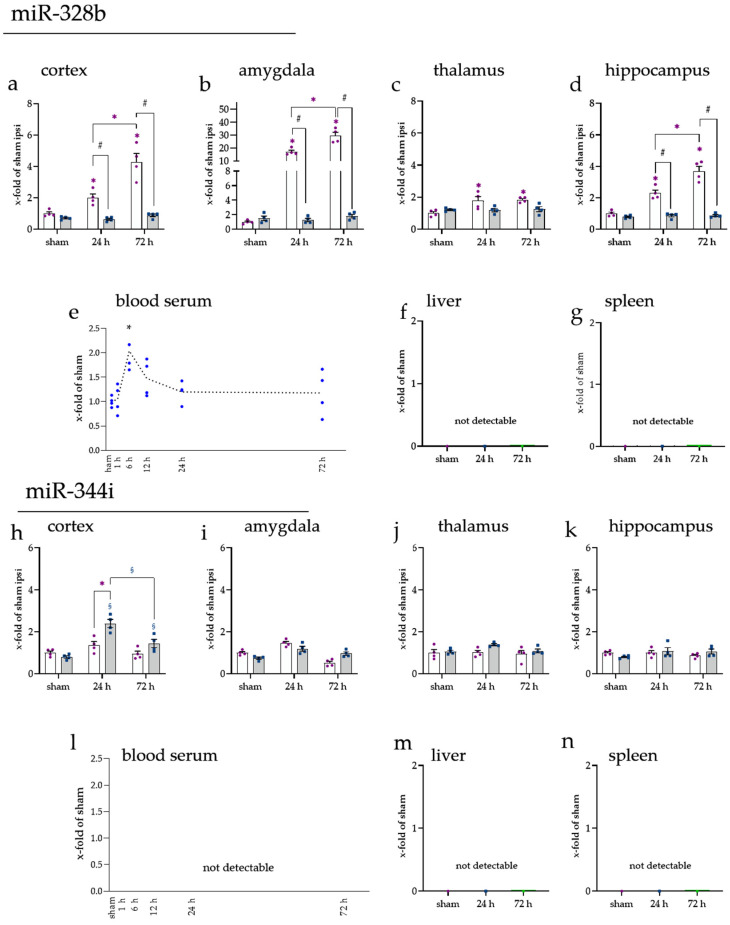
Expression of brain-enriched miRNAs miR-328b-3p (**a**–**d**) and miR-344i (**h**–**k**) in the investigated peri-infarct area and non-affected brain regions, (**e**) blood serum, (**f**) liver, and (**g**) spleen. In the brain, miR-328b was dysregulated in different brain regions and showed a short boost in the blood serum. Note that miR-328b-3p was not detectable in the spleen and liver. On the contrary, miR-344i showed only a slight increase in the peri-infarct area in the cortex but was not detectable neither in the blood serum (**l**) nor in the peripheral organ’s spleen (**n**) and liver (**m**). (*) shows a significant difference between the ipsilateral side and sham ipsi, where (§) shows a significant difference between the contralateral side and sham contra. (#) comparison as indicated.

As the last step, the miRNAs of the array were compared with miRNA databases, and miR-328b-3p [34,35] and miR-344i [34,35] were identified as brain-enriched among others. These miRNAs were measured in all tissues to determine a possible distribution process into peripheral organs (Figure A5). For miR-328b, we detected alterations in the cortex (peri-infarct area), amygdala, and hippocampus, as well as a short boost during the first hours of reperfusion in the blood serum. In the spleen and liver, no signal was detected. For miR-344i, alterations were detected only in the cortex, whereas no signal was detected in the blood serum or the spleen and liver.

## 3. Discussion

Using rats, we sought to investigate the post-ischemic distribution of picked miRNAs after an ischemic stroke. We looked at the affected peri-infarct area in the cortex and non-affected CNS regions such as the amygdala, thalamus, hippocampus, cerebellum, and spinal cord. Finally, peripheral organs (spleen and liver) were also investigated besides the blood serum as a potential distributing medium. The reasons for investigating the below-mentioned miRNAs were their involvement in inflammation and their regulation in the miRNA array (Figure A1; for more details, please see section “Reverse Transcription (RT) and quantitative real-time PCR (qrtPCR) (cDNA and miRNA cDNA, normalization strategy of miRNA”).

We observed that the miRNA expression increased time-dependently on the ipsilateral side of the cerebral cortex and contralateral after 24 h for miR-448-5p and 72 h for miR-223-3p. These alterations seem to be primarily independent of proteins that perform miRNA biosynthesis, although a slight increase in *Dicer* and *Ago* was observed after 24 h in the cerebral cortex (Figure A3). In one of our previous studies, we found elevated Ago and Dicer proteins after oxygen–glucose deprivation (OGD) using the human microglial cell line HMC-3 [36]. After observing the alterations in miRNA expression on the contralateral brain hemisphere, we wanted to assess the range of miRNA alterations in the animals. Therefore, we also studied miRNAs’ expression levels in the blood serum and the peripheral organs spleen and liver. For miR-223-3p, the expression level in the blood serum rose after the onset of the injury and a similar pattern was also visible in the spleen. The expression levels of miR-155-5p dropped in the serum right after injury onset, and a similar pattern was observed in the liver.

The upregulation of the anti-inflammatory miR-223 is in line with previous results where the levels rose until 72 h in the cerebral cortex of rats after ischemic stroke [37] and were elevated in human blood samples 72 h following stroke [38,39]. The expression of miR-223 can be additionally altered depending on the comorbidity. For example, downregulation of miR-223 was observed in patients additionally suffering from hyperglycemia [40]. The pro-inflammatory miR-155 was upregulated in rodents after 24 h and in human blood, while a knockdown inhibited the inflammatory reaction [41,42]. These findings go along with our results. For miR-3473, one other study reported deregulation after ischemic stroke in rats. The levels showed a peak at 24 h and decreased afterward [43]. In mice, miR-3473b—which is almost sequence-identical to miR-3473 in rats (with three additional nucleotides)—was upregulated and contributed to inflammation [30]. Contrary to our results, no increase at 6 h was observed. For miR-448, no alteration in the brain following stroke was reported, nor a connection to ischemic stroke and blood levels. A study utilizing a spinal cord ischemia/reperfusion injury model reported increased levels of miR-448 measured by qRT-PCR [44] and in cell hypoxic culture models of myocardial infarction [31,45]. The studies, as mentioned above, on miRNA alteration in ischemic stroke have either been performed in the cerebral cortex, the blood serum, or the researchers did not indicate the exact location. In general, miR-223 and miR-155 are present in different brain regions and organs [46,47,48,49]. For miR-3473 and miR-448-5p, little is known, though miR-448 seems to improve memory impairment in the hippocampus in rats [50]. We found miR-223, -155, -3473, and -448 in all different brain regions and, except for miR-448, also in the spleen and liver. The absence of miR-448 in these organs is partly contrary to previous studies, showing that miR-448 plays a role in hepatocellular carcinoma, though this might be due to the different conditions in cancer, thus different regulation/transportation of the miRNA itself [51,52,53]. A recent project sequenced mouse and human tissues for cell and organ specificity [34]. The microarray in the present study was performed with samples only from the brain, making it impossible to compare the specificity between the different tissues. We have used the previously mentioned project to compare the miRNAs of our array for tissue enrichment. Brain-specific miRNAs could clarify if the miRNAs found altered in the peripheral organs are due to transportation or regulation procedures.

Whether the alterations in miRNA after stroke have a biological impact needs further clarification because, during the transcription, maturation, and translational inhibition, miRNA can be regulated and influenced in many different ways [54]. For example, during biosynthesis, RNA-binding proteins interact with miRNA proteins such as Drosha, Dicer, or the RISC complex, resulting in an increased level of mature miRNAs [55,56]. However, the mature miRNA can be prevented from binding correctly. A single miRNA binds to mRNA via a short seed sequence. Not only the proportion of miRNA to target is important, but there are also possible competing partners for specific binding spots [54]. In addition, the miRNA itself can be “sponged” by an endogenous competing RNA, thereby no longer working as a translational inhibitor [57]. Depending on the kind of regulation that happens to the miRNA, the most known function of repression can be reversed. Then, binding to the mRNA sequence can lead to an upregulation rather than downregulation [58,59]. Besides regulatory mechanisms to the miRNA, migrating factors could also contribute to the altered miRNA levels we observed in this study.

Following the primary injury, the inflammatory response leads to an infiltration of a range of inflammatory cells. We observed an increase in miRNA, especially miR-223, after 72 h, which could be connected to the infiltrating cells. Immune cells such as monocytes, macrophages, and granulocytes have been shown to contain high levels of miR-223 [60,61,62]. Additionally, Calvente et al. showed that infiltrating neutrophils in the liver influenced the state of macrophages via miR-223 [63]. In addition, in spinal cord injury, miR-223 was found in neutrophils and connected to increased levels of inflammatory cytokines [64]. A further explanation for elevated miR-223 levels could be platelets, which might be a source for this miRNA. Platelets promote a phenotypic switch in arterial injury repair, which is a part of restoring the blood–brain barrier after an ischemic insult [65,66]. To clarify, if the miRNA-223 levels result from infiltrating neutrophils, fluorescence-activated cell sorting (FACS) should be performed, followed by a cell-specific miRNA analysis. In an earlier FACS study of our group, especially CD45^+^ and CD45^+^CD11b^+^CD11c^+^ cell percentages increased after 72 h [67]. These cell markers of the cluster of differentiation are used to identify the phenotype of immune cells. CD45 is carried by leukocytes, while CD11b and CD11c are also found on monocytes, macrophages, and granulocytes. In addition, miR-155, which we found to be elevated after 24 h and 72 h, was previously found in maturing dendritic cells [68], monocytes, and macrophages [69,70,71], as well as in the microvesicles of neutrophils where it contributed to NF-κB activation [72,73]. The lymphatic B- and T-cells have also been shown to contain miR-155 and miR-448-5p [71,74]. Mir-448 levels have been shown to increase upon Il1β stimulation via the NF-κB pathway [74]. This pathway is part of the immune response after tMCAo [75]. The idea of infiltrating cells might be supported by the fact that we could not detect brain-enriched miRNAs, such as miR-328b [34,35] or miR-344i [34,35], neither in the spleen nor in the liver (see Figure A5), despite an initial boost of miR-328b in the blood serum. These results do not support the theory of miRNAs being released, neither active nor passive, to function as communication signals or triggers in other regions following AIS. However, we suppose that, in the case of an active release, specific miRNA candidates might be extruded and suited as a trigger and other miRNAs, such as these aforementioned brain-enriched miRNAs, might not. Therefore, the source of the regulated miRNAs in stroke-independent regions remains indefinite for now and further investigations are needed to clarify this issue.

Infiltrating immune cells could also contribute to the significant changes measured in the amygdala since it is also supplied by branches of the anterior choroidal artery (ACHA) from the internal carotid artery, just as the cerebral cortex [76]. The myeloid and lymphatic cells being part of the immune response following ischemic stroke could contribute to the elevated levels measured in this study. On the other hand, the origin of the ACHA might also be occluded by the filament during the tMCAo procedure, since it is located near the origin of the MCA [77]. The supplied areas in rats are mainly the cortex, striatum, thalamus, and hypothalamus [78]. The blood flow to the hippocampus in rodents is primarily ensured by the anterior choroidal artery and posterior hippocampal artery; additionally, it can be impaired [79]. Moreover, the extent of the infarction can be influenced by the kind of inserted filament and the duration of the occlusion [80,81]. Interestingly, when we assume high similarities between the mouse and rat brain blood supply, thalamic branches also arise from the ACHA [82], whereas, in the thalamus, almost no changes were detected.

*Nlrp3, Socs1, Socs3*, and *Vegfa* were measured as target genes of miR-223-3p, miR-155-5p, miR-3473, and miR-448-5p, respectively. All presented an upregulation following ischemic stroke in the cerebral cortex and other brain regions to a different extent. Interestingly, regions that are distant from the original injury site also showed a regulation at a crosstalk phenomenon. Measurements besides the actual injury area are lacking for all four proteins. It was previously described that NLRP3 is highly upregulated following AIS [83], contributing to the inflammatory reaction, and that, in turn, the inhibited inflammasome reaction led to a better outcome [84]. Unknown was the regulation of *Nlrp3* in the amygdala, spleen, and liver. A review of central poststroke pain (CPSP) discussed the influence of NLRP3 on the thalamus and supposed that the inflammasome might contribute to CPSP by inducing thalamic lesions [85]. However, the regulation in the amygdala and thalamus was visible after 72 h, a later time point, suggesting that consecutive reactions and the release of DAMPs could lead to this upregulation in stroke-independent regions. The SOCS proteins regulate cytokine responses in the CNS, resulting in a limited inflammatory response [86], while, especially for SOCS3, competing effects have been described due to numerous known interactions in the CNS following acute or chronic diseases [86]. An in vitro study in the murine microglial cell line BV-2 found SOCS1 increased upon oxygen–glucose deprivation [87]. Contrary to this and our study, no alteration following tMCAo was observed in a mouse model of AIS in the cerebral cortex [88]. SOCS3 displayed a tremendous increase in all four target genes following ischemic stroke, as described before [89,90]. One study found the reactive astrocytes are responsible for the elevated SOCS3 levels in the hippocampus following an ischemic insult in rats. VEGFA is a known brain marker for brain swelling following AIS and is upregulated in edema-rich regions [91]. When we take the expression results of the target genes of the picked miRNAs into consideration, which were altered in the investigated brain regions and spleen and liver, we assume that, somehow, a trigger as a communication signal might be released on purpose or accidentally to induce gene expression changes in the investigated non-affected regions after ischemic stroke.

This study focused on the miRNA expression in different brain parts, serum, and organs without respect to the individual cell types. It would be interesting to see how the miRNA levels are correlated to specific cell compositions. A connection of the ipsi- and contralateral hemisphere is the corpus callosum. It has been shown that cells migrate along the corpus callosum to reach the ipsilateral side following stroke injury [92]. Are the alterations based on migration or regulation of miRNA levels during transcription or maturation? Further, the major downside of the study is its descriptive nature and focus on the expression of miRNAs and their selected target genes. However, our results hint at a systemic influence of AIS at different levels, but no conclusion of a direct influence of miRNAs on their target mRNA can be drawn. Further interaction studies, also on protein level, would be needed. Nevertheless, regarding our central hypothesis, if we can find changes in miRNA expression patterns in non-affected brain regions and peripheral organs, we conclude that our results indicate a possible interaction between the affected peri-infarct zone and specific brain regions, as well as peripheral organs. A deeper investigation will be the next step to clarify the possibilities of such interactions and how they might work.

For future studies, it would be interesting to identify the pathways that lead to an increased gene expression of miRNA to better understand the working mechanism following injuries such as ischemic stroke.

## 4. Materials and Methods

### 4.1. Animals

The Review Board for the Care of Animal Subjects of the distinct government (North Rhine-Westphalia, Germany, 84-02.04.2013.A212) approved the experiments and animals for this study. Male Wistar rats (8–10 weeks old) with an average weight of 300–350 g were used (Janvier Labs, Le Genest-Saint-Isle, France). After delivery, animals could adapt to the new animal housing for one week before starting the experiments. The rats were kept under a 12 h light/dark cycle with food and water ad libitum. The hygiene management and caretaking of the animals were performed following the guidelines and microbiological monitoring of the Institute for Laboratory Animal Science and Experimental Surgery (University Hospital Aachen, RWTH Aachen University) according to the FELASA recommendations. The animals were randomly assigned to their groups (*n* = 4) and operated accordingly. After the operation, animals were housed individually and strictly monitored until the time point of their sacrifice. For the sham group, only 24 h was used as a reference based on the three R concepts (replacement, reduction, and refinement) in animal studies. Beforehand, no variations of the sham groups were observed over the time of the experiments [93].

### 4.2. Transient Middle Cerebral Artery Occlusion (tMCAo)

As previously described, a middle cerebral artery (MCA) occlusion was performed [93]. In brief, animals were anesthetized with 2–3 vol% isoflurane (Abbott, Wiesbaden, Germany) and maintained at 1–2 vol% during the procedure (60 min occlusion and 30 min operation). For the analgesia, 0.01 mg/kg Temgesic was applied 1 h before the operation. During the operation, the body temperature was controlled and eye ointment was applied. A sufficient MCAo was monitored by the reduction in the cerebral blood flow using a laser-doppler (Moor Instruments VMS-LD2, Millwey, UK). To temporarily block the blood flow in the MCA, an incision in the common carotid artery was performed and a polyamide thread with a silicone cap (Doccol Corporation, Sharon, MA, USA) was inserted until the branching of the internal carotid and middle cerebral arteries. After 60 min occlusion, the filament was carefully pulled back, and the wounds were closed. For sham surgery, everything was performed similarly. Only the filament was not threated forward to occlude the MCA.

### 4.3. Neurological Evaluation

Neurological deficits were assessed to control for sufficient stroke symptoms as described before [93,94]. Here, the motor and sensory abilities of the animals were tested, and up to three points were given if the animal was able to perform the task. The activities analyzed were spontaneous activity, forepaw outstretching, climbing, body proprioception, spontaneous walking activity, and sensory function. Two researchers scored the animals in a blinded manner before they were sacrificed.

### 4.4. Finalization and Tissue Sampling

At the designated time point after the operation, animals were deeply anesthetized with Ketamine/Xylazine (100 mg/kg and 10 mg/kg i.p.). Blood samples were taken before the animals were transcardially perfused with ice-cold PBS. Collecting tubes containing a coagulant were used to retrieve the serum (Sarstedt AG & Co.KG, Nümbrecht, Germany). The collected blood was incubated at room temperature for 15 min and was then centrifuged (15 min, 4 °C, 3000× *g*) and stored. The brain was removed and cut into slices of 2 mm using a brain matrix. The brain slices were stained with 2% TTC (2,3,5-Triphenyltetrazolium chloride) for 15 min (37 °C, 55 rpm) and photographed with a squared paper in the background for later analysis of the infarct size. TTC visualizes tissue viability through an enzymatic reduction to a red formazan product [95]. The brain samples were punched out from the peri-infarct zone with a 2 mm punch (Figure A1c). The liver sample was taken from the left lobe, and a lateral part of the spleen was collected. All tissue samples were snap-frozen in liquid nitrogen until later use.

### 4.5. Edema-Adjusted Infarct Volume (EAIV)

During tMCAo, edema appears on the ipsilateral side of the brain, which must be included in the calculation and corrected [96]. To determine the infarct volume, the area size of the infarct (aIF), the whole brain slice (atb), and the ipsilateral hemisphere (ai) were measured and calculated together, also considering the 2 mm thickness of the slice. In the end, the results from every slice were summed up. Therefore, the final volume was referred to edema-adjusted infarct volume (EAIV). The measurements of each area were performed using ImageJ [97].

(1) $$\mathrm{EAIV}=(a_{\mathrm{IF}}*\;\frac{\left(a_{\mathrm{tb}}-{\;a}_{i}\right)}{a_{i}})*2\;\mathrm{mm}$$

### 4.6. RNA Isolation

Two different approaches were performed to measure the miRNA expression in tissue and blood serum. The whole RNA was isolated from the tissues using a phenol-chloroform method (TriFast, PeqLab, Erlangen, Germany). Following the manufacturer’s protocol, samples were first homogenized, then chloroform was added. After centrifugation, three phases were visible and the RNA-containing upper phase was used for further processing. Isopropanol was added to precipitate the RNA, which was pelleted and washed. After resuspension in ultra-pure water, concentration and purity were measured using the nanodrop (PeqLab, Erlangen, Germany). Samples were used only when the quality ratios of A_260_/A_280_ were higher than 1.9 and A_260_/A_280_ were higher than 2.1.

The same amount of serum from all animals was used for serum isolation. A spike-in approach was adopted for serum isolation using cel-miR-39 (UCACCGGGUGUAAAUCAGCUUG, Thermo Fisher Scientific, Waltham, MA, USA) which was added to the samples before RNA isolation. First, the serum samples were supplemented with Trifast (PeqLab, Erlangen, Germany); then, the cel-miR-39 was added in the final concentration of 28 pmol per sample. After adding chloroform, the RNA-containing upper phase was used to isolate the RNA described above. Finally, after the cel-miR-39 was detected in all serum samples, it was used for normalization.

### 4.7. MicroRNA Profiling Using Affymetrix miRNA 4.0 Arrays

GeneChip^®^ miRNA 4.0 Array (ThermoFisher Scientific, Waltham, MA, USA) was used to analyze three independent experiments per group. The investigated groups were sham ipsi and sham contra, and tMCAo ipsi and tMCAo contra. For each subject, 1.5 µg of total RNA containing low molecular weight RNA was labeled using the FlashTag™ Biotin HSR RNA Labeling Kit according to the manufacturer’s instructions (ThermoFisher Scientific, Waltham, MA, USA).

Briefly, for each sample, 1.5 µg total RNA was subjected to a tailing reaction for 15 min at 37 °C (2.5 mM MnCl_2_, ATP, Poly A Polymerase), followed by ligation of the biotinylated signal molecule to the target miRNA sample for 30 min at RT (1 x Flash Tag ligation mix biotin, T4 DNA ligase), adding the appropriate stop solution. Each sample was hybridized to a GeneChip^®^ miRNA 4.0 Array for 18 h at 48 °C and 60 rpm (Affymetrix, Santa Clara, CA, USA). Afterward, the chips were washed and stained on Fluidics Station 450 (Fluidics script FS450-0002) and scanned on a GeneChip^®^ Scanner 3000 7G (both, Affymetrix, Santa Clara, CA, USA). Data were analyzed using expression console software 1.4 (Affymetrix, Santa Clara, CA, USA). The expression values were normalized with a robust multi-array average (RMA) [98]. miRNA calculations and statistical significances between two groups (paired Student’s *t*-tests) were performed with Transcriptome Analysis Console (TAC) Software (ThermoFisher Scientific, Waltham, MA, USA). Detected miRNAs whose expression levels were significantly different (*p* < 0.05) and showed a linear-fold change of at least 1.5 compared to the control group (sham ipsi) were considered as differentially expressed. Graphpad Prism software (Version 9.0.0, macOS version) was used to design the heat map showing intensity variability between biological replica (with a 2-fold cut-off and an adjusted *p*-value of < 0.05; *n* = 3; hierarchical clustering heat map using cosine column and row clustering, rows were normalized relative to median).

### 4.8. Reverse Transcription (RT) and Quantitative Real-Time PCR (qrtPCR) (cDNA and miRNA cDNA, Normalization Strategy of miRNA)

After total RNA isolation, two kinds of RTs were performed, one specific for small RNAs and one for all types of RNA. The first one served for miRNA studies, while the second was used for cDNA of genes regarding the maturation of miRNAs and the targets *Nlrp3, Socs1, Socs3, and Vegfa.*

The RT for small RNAs was performed according to Busk et al. [99]. With a poly(a) polymerase (M0276L, New England Biolabs, MA, USA), polyadenylation to the miRNA strand was performed and a set of random primers (CAGGTCCAGTTTTTTTTTTTTT-TTVN) was used to perform the reverse transcription using M-MuLV reverse transcriptase (M0253L; New England Biolabs, MA, USA). After the reverse transcription, a check PCR was performed using *U6* to control for adequate transcription in all tissue samples. The check PCR was performed with suitable primers for cel-miR-39 (spike-in, Table 1) for serum samples. miRNA expression was determined by a semi-quantitative real-time PCR (qrtPCR) using SYBR green (BrightGreen 2x qrtPCR MasterMix, Applied Biological Materials Inc., BC, Canada). Relative quantification was calculated using the ΔΔCt-method with the applied Bio-Rad software [100]. The running protocol for miRNA was conducted as follows: 95 °C for 10 min enzyme activation, a 40x repetition cycle of 95 °C for 3 s for denaturation, and T_A_ (specific annealing temperature for each primer) for 20 s for annealing and elongation. Finally, a melting curve was made to control a clean amplicon. An agarose gel electrophoresis was performed to control that no side product occurred. To normalize miRNA qRT-PCR data, it is essential to find the right normalization strategy to overcome technical-based bias [101]. In the blood serum, the spike-in cel-39 was used as a reference. For the other tissues, several miRNAs were tested for their stability using Bio-Rad CFX manager 3.1. Primers were used with a recommended coefficient variance <0.25 and an *M*-value <0.5 [102]. Three had a stable expression pattern over all experimental groups, i.e., snoRNA U73, miR-103, and miR-107. Using multiple miRNAs for normalization stabilizes the results [102,103].

We have picked four miRNA candidates with a stable expression and low standard deviation in qRT-PCR for expression studies within the brain and peripheral organs. Further selection criteria were based on literature research. In our group’s previous study, miR-223-3p levels were examined following stroke with estrogen and progesterone treatment [37]. Thereby, miR-223-3p was already identified as a promising candidate for further studies. For miR-3473, only two references were found in Medline in combination with ischemia at the beginning of the study [30,43]. Since inflammation plays a significant role following AIS, we were interested in the pro-inflammatory miRNA-155-5p [26]. The last miR-448-5p was chosen because it showed the highest reduction after tMCAo in the array.

For the cDNA synthesis of all RNA molecules, 500 ng of total RNA was used. The RT was performed using the SensiFAST^TM^ kit (Bioline, London, UK) following the manufacturer’s protocol. The success was checked with gel electrophoresis using cyclophilin A (Cyclo A) primer. Again, a qRT-PCR was performed using the following protocol: 95 °C for 2 min, 40 cycles of 95 °C for 5 s, and T_A_ for 25 s, followed by a melting curve. An agarose gel electrophoresis was performed to control for unwanted side products. All primer sequences used in this study can be found in Table 1. Relative quantification was calculated using the ΔΔCt-method with the applied Bio-Rad software [100]. Data are expressed as the relative amount of the target to the amount of 2 housekeeping genes, namely, CycloA and Glycerinaldehyd-3-phosphate dehydrogenase (GAPDH).

### 4.9. Statistics

Graphpad Prism 8.3.0 (GraphPad Software Inc., San Diego, CA, USA) was used to calculate the statistics of the data and make the graphical representation of the figures. All data are represented either as individual data points or mean ± SEM. Normality of residuals was tested either using the Shapiro–Wilk (one-way ANOVA) test or Spearman’s test for heteroscedasticity (two-way ANOVA). If one test was significant, a box-cox transformation was performed. A two-way ANOVA with Tukey’s post hoc test was performed for the brain samples, including the comparison of ipsi- and contralateral brain hemispheres and the different time points. A Brown–Forsythe test for variance homogeneity was performed for the spinal cord, serum, spleen, liver, and EAIV. Then, a one-way ANOVA with Tukey’s post hoc test was performed to compare the time points. The numbers of animals used in the study and the biological samples are summarized in Figure A2a. Individual symbols indicate significant differences regardless of the significance level (0.05, 0.01, or 0.01).

## 5. Conclusions

In summary, we observed alterations in the expression levels of miR-223-3p, miR-155-5p, miR-3473, and miR-448-5p in different locations of the brain, serum, spleen, and liver after tMCAo in rats. All miRNAs changed their expression in both the ipsi- and contralateral hemispheres in a time-dependent manner after cerebral stroke, especially in the cerebral cortex and amygdala. This points to a transportation or signaling event for the miRNAs within the brain or a local enrichment in inflammatory cells with an associated miRNA content. The expression levels of miR-223-3p were elevated in the blood serum and spleen shortly after reperfusion. For miR-155-5p and miR-3473, a drop in the serum was observed.

Additionally, altered expressions of the selected miRNAs in peripheral organs, which were not affected by the ischemic insult, indicate possible crosstalk between the CNS and peripheral systems. Further studies should focus on tracing miRNAs to track the course of the miRNA and to figure out the potential of such crosstalk between the CNS and the periphery. Identifying specific targets in the peripheral organs could help define the influence of such expression changes and might lead to new ideas in developing stroke treatment approaches.

## Figures and Tables

**Table 1:** List of primers used in this study.

Primer Name	Sense	Anti-Sense
*U6*	CCCTGCGCAAGGATGA	AGGTCCAGTTTTTTTTTTTTTTTAATTTG
cel-miR-39	GTCACCGGGTGTAAATCAG	GGTCCAGTTTTTTTTTTTTTTTCAAG
miR-103	GCAGAGCAGCATTGTACAG	GGTCCAGTTTTTTTTTTTTTTTCATAG
miR-107	GCAGAGCAGCATTGTACAG	GGTCCAGTTTTTTTTTTTTTTTGATAG
miR-223-3p	CGCAGTGTCAGTTTGTCA	CCAGTTTTTTTTTTTTTTTGGGGTA
miR-155-5p	CGCAGTTAATGCTAATTGTGATAG	AGGTCCAGTTTTTTTTTTTTTTTACC
miR-3473	CAGTCTAGGGCTGGAGAG	CCAGTTTTTTTTTTTTTTTAGCCATC
miR-448-5p	CGCAGAACATCCTGCATAG	GTTTTTTTTTTTTTTTGGCAGCAC
miR-328b-3p	GCTGGCCCTCTCTGC	AGGTCCAGTTTTTTTTTTTTTTTAGG
miR-344i	CTCTAGCCAGGGCTTGA	TCCAGTTTTTTTTTTTTTTTGCAGT
*cyclophilin A*	GGCAAATGCTGGACCAAACAC	TTAGAGTTGTCCACAGTCGGAGATG
*Gapdh*	AACCCATCACCATCTTCCAG	GTGGTTCACACCCATCACAA
*Ago*	CCCCCACCTCCCATGTTTAC	GACCTGGCAGTTGCTCTGAT
*Dicer*	GAAGAGGAGACCAGCGTTCC	CGGGTTTGGGGTAACTCTCC
*Drosha*	CTGGGACGAAACCAAGCTCT	CATAACTCAACTGTGCAGGGC
*Trbp*	GGATCATGGCCGGTAGCAAA	CTAGGCAGACGATAGACCC
*Dgcr8*	GCGAAGAATAAAGCTGCCCG	TTGTCAGCTCGTAGACTCGC
*Xpo5*	AGAATCTGGTCGCTTGGTGG	CCGTCAGAAGGGCAAGATGT
*Nrlp3*	TCTGTTCATTGGCTGCGGAT	GCCTTTTTCGAACTTGCCGT
*Socs1*	CCGCTCCCACTCTGATTACC	CTCAGGGGTCCCCAGTAGAA
*Socs3*	CGACGGAACCTTCCTTTGAGG	AGAGGTCGGCTCAGTACCAG
*Vegfa*	ATCTTCCAGGAGTACCCCGAT	CGCATGATCTGCATAGTGACG

## Data Availability

Datasets generated and analyzed during this study are available from the corresponding author upon reasonable request.
